# Valve-in-Valve Challenges: How to Avoid Coronary Obstruction

**DOI:** 10.3389/fcvm.2019.00120

**Published:** 2019-08-23

**Authors:** Fernando L. M. Bernardi, Danny Dvir, Josep Rodes-Cabau, Henrique B. Ribeiro

**Affiliations:** ^1^Hospital São Francisco-São Camilo, Concórdia, Brazil; ^2^Heart Institute of São Paulo (InCor), University of São Paulo, São Paulo, Brazil; ^3^Division of Cardiology, University of Washington, Seattle, WA, United States; ^4^Department of Cardiology, Quebec Heart and Lung Institute, Laval University, Quebec City, QC, Canada

**Keywords:** valve-in-valve, transcatheter aortic valve replacement, coronary obstruction, failed surgical bioprosthesis, transcatheter heart valve

## Abstract

Coronary obstruction is a rare but life-threatening complication in patients undergoing transcatheter aortic valve replacement (TAVR). Aortic valve-in-valve (VIV) procedures to treat failed surgical bioprosthesis is associated with ~6-fold higher risk for coronary obstruction in certain situations. The primary mechanism consists in the occlusion of the coronary ostium by the dislodged leaflet from the bioprosthesis after deployment of the transcatheter heart valve (THV), which most commonly occurs during the index procedure, but in up to 1/3 of cases a delayed presentation ensues. The clinical presentation consists of severe hypotension and ECG changes in most of the patients, with very high mortality rates. Therefore, pre-procedural multi-slice computed tomography is crucial for identifying high-risk features, such as low coronary heights, shallow sinuses of Valsalva, and short virtual THV to coronary ostial distance (VTC). Also, some models of surgical bioprosthesis present an increased risk for this dreadful complication. Preemptive protective strategies with coronary wiring, with or without placement of an undeployed stent, could mitigate the risks associated with this complication in high-risk patients, even though studies are lacking. This review aims to take a clinical perspective on the challenges in avoiding this complication during VIV procedures.

## Introduction

Transcatheter aortic valve-in-valve (VIV) replacement has become an attractive and feasible treatment option for patients with failed aortic bioprosthetic surgical heart valves (SHV) in order to avoid a redo operation (1–3). More recently, VIV has also been shown suitable for patients with failed aortic transcatheter heart valves (THV) (4, 5). Nonetheless, despite excellent results being reported in recent studies, in comparison to transcatheter aortic valve replacement (TAVR) for native aortic valve disease, patients undergoing VIV procedures show lower device success rates with higher residual transaortic gradients and higher intraprocedural complications (6, 7). Fortunately, more contemporary data has shown better results regarding the rates of procedural complication and mortality, most probably due to better patient selection, improvements in THV technology, and in many procedural steps (8).

**Figure 1:**
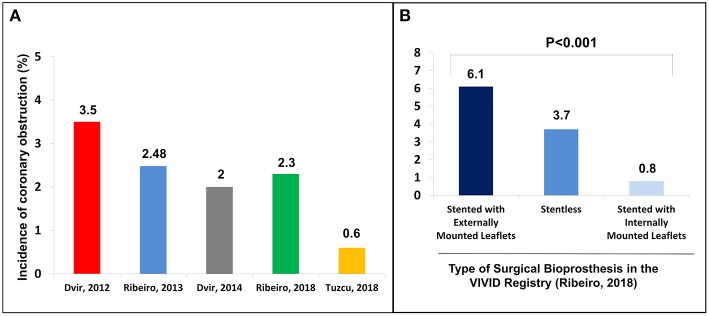
**(A)** Incidence of coronary obstruction in the main studies in the context of valve-in-valve; **(B)** Incidence of coronary obstruction according to the type of surgical bioprosthesis. Adapted from Ribeiro et al. (10). Copyrights (2017), with permission from Oxford University Press (license number: 4586671024004).

Among the complications related to the procedure, coronary obstruction stands out as one of the most worrisome during aortic VIV (9). Studies to date have shown an incidence of up to 3.5% of coronary obstruction in aortic VIV, which is ~4- to 6-fold higher than what has been reported for TAVR in native aortic valve (3) (Figure 1). In fact, VIV is considered an independent risk factor for coronary obstruction (11). Furthermore, despite the low absolute frequency of its occurrence, acute obstruction of a coronary ostium—which in more than 90% of the cases involves the left main coronary artery—is associated with very high mortality rates (10). In this article, we will review the challenges to avoid this dreadful complication in transcatheter aortic VIV.

## Mechanism of Coronary Obstruction and Risk Factors

The primary mechanism involved in coronary obstruction during VIV is related to dislodgement of the SHV bioprosthetic leaflets toward the coronary ostium as a consequence of the THV expansion (10). Less frequently, the event can occur when the bioprosthesis structures extend above the sinotubular junction (STJ), keeping close contact with the aortic wall. Therefore, when the THV is deployed the leaflets of the previously implanted SHV creates a covered cylinder in the initial portion of the ascending aorta. Understanding those mechanisms are crucial to comprehend the main risk factors associated with this complication.

**Figure 2:**
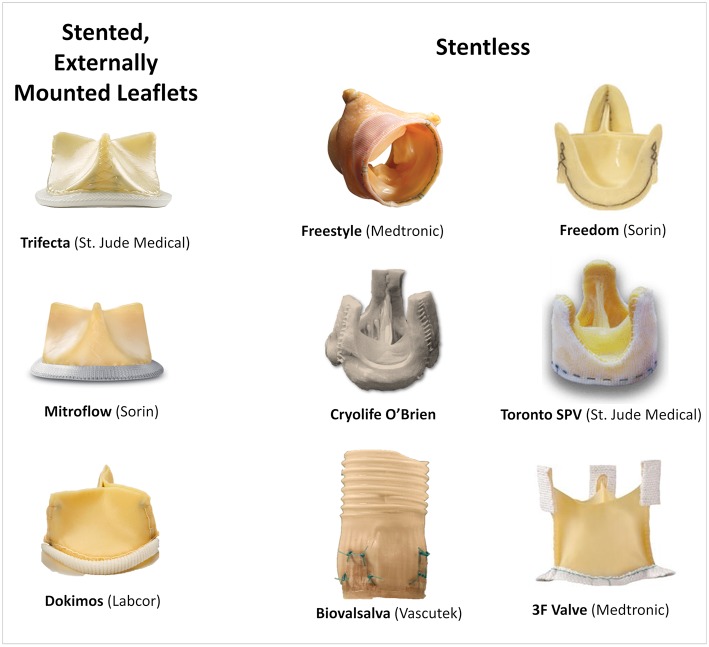
Main surgical bioprosthesis related with an increased risk of coronary obstruction.

Coronary height, defined as the distance from the coronary ostium to the aortic valve annulus, is one of the most important risk factors for coronary obstruction in native valve TAVR (11). In such, a cut-off of 12 mm for both right and left coronaries have been established as a risk factor (11). Patients with SHVs more often present with lower coronary heights in comparison to native valve TAVR cases, as frequently the SHVs are sutured in a supra-annular position (12). Of note, during VIV procedures the coronaries origin should be measured in relation to the sewing ring or the basal plane of the bioprosthesis. Nevertheless, for VIV a clear cut-off value has not been well-defined since other factors related to the type of SHV and surgery performed can be more determinant for the risk of coronary obstruction. For instance, stentless and stented bioprosthesis with externally mounted leaflets have a higher chance of provoking a more intense interaction between their leaflets and the coronary ostium during a VIV procedure, because they are supra-annular, their leaflets tend to extend outward beyond the frame of the device once the THV is expanded, and the leaflets are longer. The most frequent SHVs related with increased risk of coronary obstruction are shown in Figure 2.

In addition, shallow SOV and low STJ are other key anatomic aspects that can increase the risk of obstruction. Patients with large SOV present more room to accommodate the dislodged leaflet without compromising the coronary ostia. There are situations though, where the surgeon needs to reimplant or reconstruct the aortic root during a surgical aortic valve replacement, which may distort the anatomy and further approximate the SHV to the coronary ostium. Another vital factor to be observed is the angulation in which the SHV was mounted in relation to the axis of the ascending aorta. A bioprosthesis might obstruct a coronary during VIV even in wide SOV (13). Finally, with regards to delayed coronary obstruction (DCO), defined by obstruction occurring after the procedure, although rare, it can occur more frequently during VIV procedures and with self-expandable devices, which tend to keep expanding within hours/days after the procedure (10, 12, 13). Also, a total of 59.3% of DCO events (*n* = 16 from 27 with CT scan data available for central analysis) occurred in patients with a ≤ 3-mm difference between mean SOV diameter and size of valve implanted, highlighting this measurement as a possible risk factor (14).

## Clinical Presentation

In the largest cohort of patients with coronary obstruction following VIV, more than half of the cases (58.3%) developed severe persistent hypotension. ECG changes were detected in 52.8% of the time, with ST-segment elevation being the most common finding (68.4%), followed by ventricular fibrillation (21.1%). These high rates of severe clinical presentation are explained by the fact that in 91.6% of the cases, the left coronary artery was involved. Most often the event took place during the index procedure (63.9%), but the obstruction occurred within 24 h and more than 24 h following the VIV in 22.2 and 13.9% of the patients, respectively (10). Thus, if immediately after valve implantation ECG changes and/or severe persistent hypotension occur, coronary obstruction as a differential diagnosis should be excluded, by angiography, either with aortography or selective coronary catheterization, or new segmental abnormalities in the echocardiogram.

Another recent study has specifically evaluated delayed coronary obstruction cases. Among 17,092 patients, an incidence of 0.22% was found (38 cases), and it occurred five times more frequently in VIV procedures. Delayed cases most likely occured within 7 days after the TAVR procedure (63.2%; *n* = 24), the majority during the first 24 h (75%; *n* = 18). This should raise attention from the heart team to keep close monitoring of high-risk patients during the initial days' post-procedure, even in uneventful cases (14).

## How to Evaluate the Risk of Coronary Obstruction

Coronary obstruction following VIV generally occurs as a consequence of a combination of factors. Among them, the two most important are the type of SHV previously implanted, and the anatomical relation between both the coronary ostia and the expected final position of the bioprosthetic leaflets that will be dislodged by the THV.

Regarding the failed SHV, the first mandatory step is to correctly identify its model and size, either by the surgery description on medical records or by the characteristics of the valve on fluoroscopy or multislice computed tomography (MSCT). In cases where the surgical description is unavailable, the angiographic appearance may easily help in identifying the exact model. An online app is available to help in both identifying the surgical bioprosthesis model and in the sizing of the proper THV (https://www.pcronline.com/PCR-Publications/PCR-mobile-apps/Valve-in-Valve-Aortic-app) (15). Recent SHV has included the label size in the frame to allow easy identification, and an expansion zone to allow for future VIV procedures. The main surgical valve models related with a higher risk of coronary obstruction are stentless valves and stented valves with externally mounted leaflets, which associate with a 3- and 6-fold increase in its risk, respectively (10) (Figures 1, 2).

Additionally, meticulous pre-procedural planning with MSCT has become the most important tool for evaluating the risk of coronary obstruction. Due to its high resolution and three-dimension imaging, MSCT enables for precise identification of the SHV and thickness of their failed leaflets, determination of the bioprosthesis angulation in relation to the aortic annulus, measurement of the coronary ostia distance to the plane of the valve, SOV size, and STJ height. However, more importantly, it is possible to determine the proximity of the coronary ostia to the anticipated final position of the displaced bioprosthetic leaflets, which forms a tubular structure together with the implantation of the THV.

**Figure 3:**
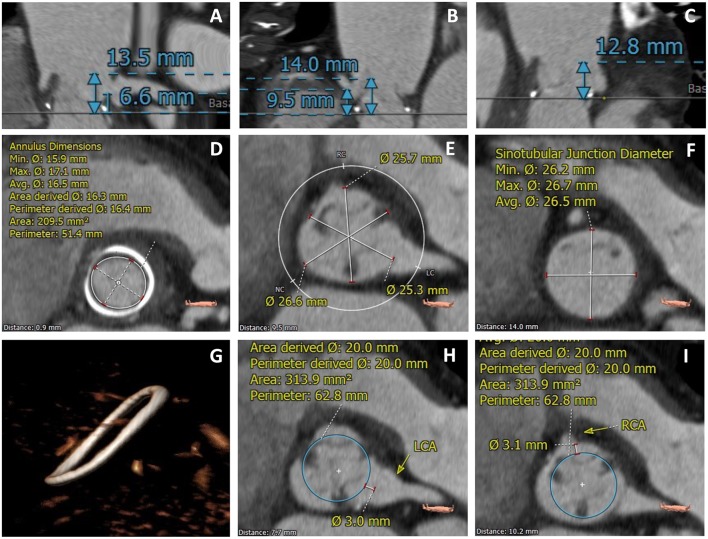
MSCT evaluation of a TAVR VIV case with high-risk features for coronary obstruction. **(A)** Left coronary height to the valve plane of 6.6 mm; **(B)** Right coronary height to the valve plane of 9.5 mm; **(C)** Low STJ; **(D)** Measurement of the SHV internal diameter; **(E)** Measurement of the SOV diameters; **(F)** Measurement of the STJ diameters; **(G)** Identification of SHV (Medtronic Hancock Standard); **(H,I)** VTC value <4.0 mm for both left and right coronary arteries.

The Vancouver approach, a method referred as virtual THV to coronary ostial distance (VTC), evaluates through MSCT imaging the distance between a virtual THV, at a size of the implanted device, toward each coronary ostium (Figure 3). To obtain the VTC, first, the basal ring plane and the geometric center of the surgical valve are identified. Then, a virtual cylinder with the estimated nominal size of the THV (i.e., a 23 mm THV leads to a cylinder with the same height of the THV and a 23 mm diameter) is placed in the middle of the basal ring. The centers of the basal ring and of the cylinder are aligned. Finally, the horizontal distance between the edge of the cylinder and the ostia of the coronaries is measured with a caliper measurement tool of the CT imaging software. The anticipated area of the THV is estimated by the circle area formula: πR^2^, where the radius (R) obtained dividing the diameter of the device by 2. Therefore, in the case of self-expanding devices, such as the CoreValve, the worst-case scenario is used (16) (Figure 3). The VTC has shown to be an independent predictor of coronary obstruction. The lower the VTC value, the higher is the risk for its occurrence, with a distance of <4 mm best predicting coronary obstruction. It is essential to highlight that when using the VTC both the coronary height and SOV width are taken together, so that the coronary height itself, for instance, is not an independent predictor for its occurrence. This is also underscored by significant collinearity between the VTC and both the coronary height and the SOV width (10).

**Figure 4:**
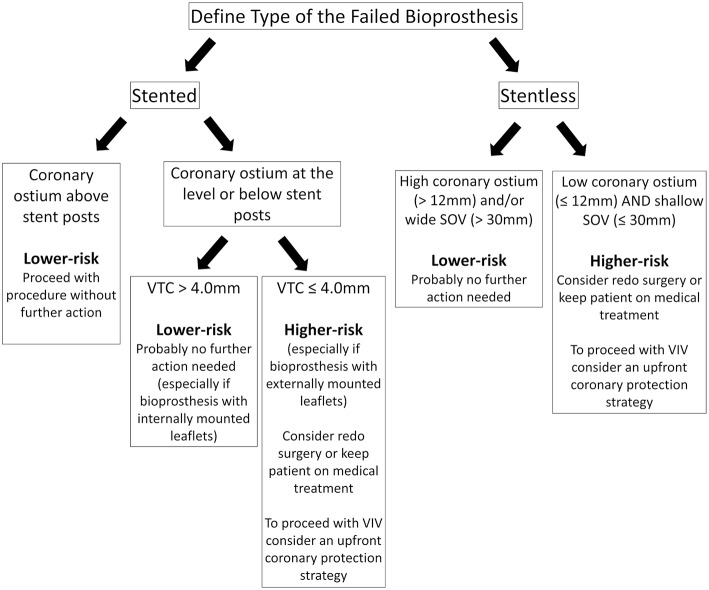
Suggested pre-procedural risk assessment algorithm for coronary obstruction in patients undergoing VIV.

The VTC method should be applied to stented SHVs when the coronary origins arise at the level or below the tip of the bioprosthesis posts. When the coronaries originate above the tip of the posts, there should be no risk for obstruction. With stentless SHVs, assessment should be similar to native TAVR (the measurement of the distance between the valve basal plane to the coronary ostia and width of the SOV). In such cases, the VTC approach is more challenging due to the lack of a basal ring or rigid scaffold from the SHV (16). Figure 4 is a suggested algorithm to assess the risk of coronary obstruction in VIV patients.

## How to Avoid Coronary Obstruction

After determining the risk of coronary obstruction during pre-procedural planning, it is crucial to delineate the strategy and how to proceed with the VIV. Low-risk patients, those with high coronary origins (take-off above the posts of stented SHVs or >12 mm from the valvular annulus of a stentless SHV), should undergo VIV without further action. If that is not the case, further evaluation should take place according to the type of SHV. In stented bioprosthesis, a VTC >4 mm reduces the risk of coronary obstruction significantly, especially with internally mounted leaflets valves, and probably no further action is needed. Likewise, in patients with stentless valves, but wide SOV and high STJ should also put the patient at low risk for coronary obstruction. On the other hand, in those with high-risk features (low coronary ostium and VTC <4 mm in stented SHVs or shallow SOV in stentless SHVs) should prompt the heart team to analyze each case individually and consider one of the following: (a) keep patient on medical treatment; (b) redo surgery; (c) proceed with VIV with an upfront coronary protection strategy.

**Figure 5:**
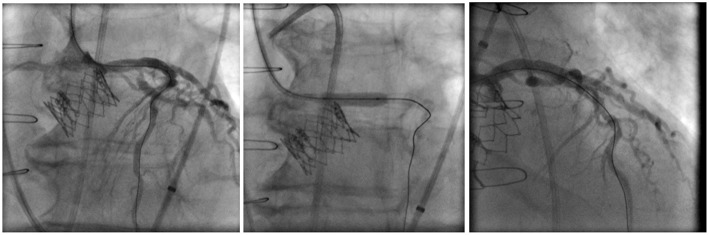
Example of a left coronary artery chimney stenting technique in a high-risk for coronary obstruction patient receiving a Sapien XT valve with a preemptive guidewire protection approach.

An upfront strategy to protect the coronaries in high-risk VIV patients is highly advisable because performing percutaneous coronary intervention (PCI) in an obstructed coronary ostium by the dislodged leaflets of an SHV can be quite challenging, especially if hemodynamic instability is present. The best technique in this scenario is still debatable, but the preemptive positioning of a guidewire and an undeployed stent could facilitate the prompt diagnosis and the treatment of a potential occlusion (17). After implantation of the THV, the patency of the coronary can be assessed by direct contrast injection through the guide-catheter, aortography, or through evaluation of any new wall motion defect with echocardiography. If a coronary obstruction is confirmed, the undeployed stent is pulled back and deployed at the ostium with some protrusion into the aorta, in what is called the chimney stenting technique (Figure 5) (18).

Another strategy is to utilize a fully retrievable or partially retrievable THVs, such as the Evolut-R, Portico, and Lotus valves, in these high-risk situations. In this approach, coronary flow status can be assessed before complete deployment of the THV, and in case of any compromise, the operator can try changing the positioning of the THV or even abandon the procedure. However, as mentioned, a coronary obstruction can occur after the VIV procedure in up to 35% of the cases, so this strategy would not be able to prevent a DCO presentation.

**Figure 6:**
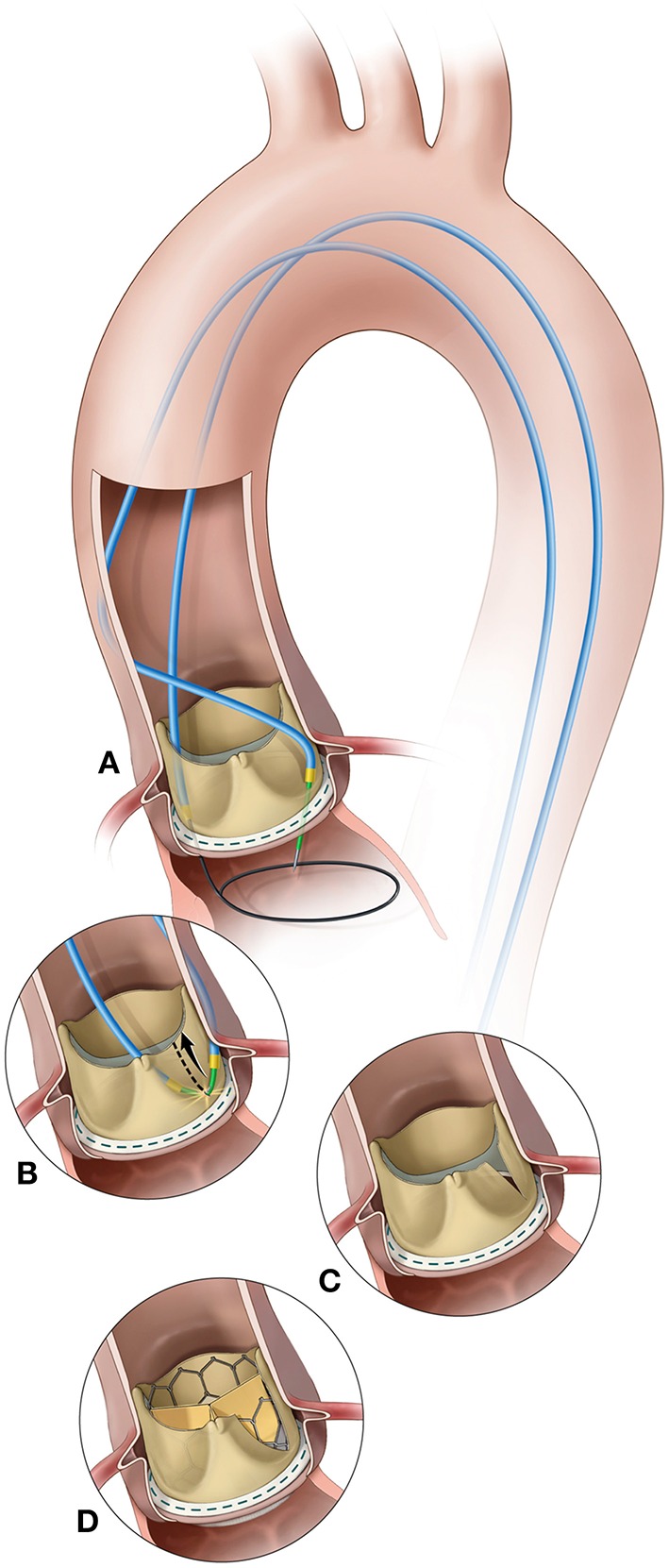
BASILICA procedure. **(A)** A catheter directs an electrified guidewire through the base of the left aortic cusp into a snare in the left ventricular outflow tract; **(B)** After snare retrieval; **(C)** The mid-shaft of the guidewire is electrified to lacerate the leaflet; **(D)** The leaflet splays after transcatheter aortic valve replacement permitting coronary flow. Reprinted from Khan et al. (19). Copyrights (2018), with permission from Elsevier (license number: 4587251472719).

More recently, a novel technique called bioprosthetic scallop intentional laceration to prevent coronary artery obstruction (BASILICA) has been described and might prove beneficial in high-risk patients undergoing VIV procedures (19) (Figure 6). In this technique, before THV implantation, the SHV leaflet that poses a risk to the coronary ostium (usually the left coronary artery) is lacerated utilizing an electrified guidewire that is punctured and snared through the leaflet. The procedure seems to be feasible in all types of SHVs and native aortic valve, but only a small series of patients have been reported. Currently, a clinical trial is ongoing to further evaluate the feasibility and safety of the BASILICA procedure in patients undergoing TAVR at high-risk for coronary obstruction (20) (ClinicalTrials.gov Identifier: NCT03381989).

## VIV for Failed TAVR Bioprosthesis

VIV for failed TAVR bioprosthesis, or redo TAVR, appears to be a feasible and safe approach, although the current evidence is limited (4, 5). Nonetheless, since the indication of TAVR is moving to lower risk patients, it is expected that TAVR degeneration requiring repeated procedures will grow considerably in the near future. To date, the largest published study evaluating this technique involved a series of 50 cases from multiple centers around the World (5). The vast majority of patients had a failed CoreValve or a first-generation Edwards-SAPIEN THV. Redo TAVR was performed with a CoreValve in 28 patients (56%), followed by SAPIEN XT in 14 (28%), SAPIEN S3 in 6 (12%), and there was only one case of Evolut R and Lotus valve each. No in-hospital death was observed. There was only one reported case of coronary obstruction that occurred by the native calcified cusp after a SAPIEN XT in a degenerated Edwards-SAPIEN valve that was resolved by urgent stenting of the left main coronary artery. In theory, after deployment of the second THV, depending on the amount of oversize, the native cusps could be further moved toward the coronary ostium or the prior THV leaflet/sealing cuff could also move in the direction of the coronary ostium. Thus, care should be taken especially in those cases where there was already a concern of coronary obstruction in the index procedure due to low coronary heights, narrow SOV, and bulky calcium in the native cusps. Perhaps the threshold for an up-front coronary protection strategy should be even lower in such cases, because of the known challenges to adequately position a guiding catheter through the implanted THV. However, further studies will be required to better assess predictive factors of coronary obstruction and the best strategy for each combination of valve types in this scenario of redo THV.

## Management of Coronary Obstruction

Coronary obstruction should be avoided at all costs or at least anticipated so that operators are prepared with an upfront coronary protection strategy. However, if coronary obstruction occurs in an unprotected scenario and there is no option for retrieving the THV, probably the first best option is to try emergent PCI and provide hemodynamic support in case of circulatory collapse. In the VIVID Registry, PCI was attempted in 77.8% of cases, being successful in only 64.3% of them. Failure of PCI was attributed to coronary cannulation failure in 30%, wire crossing failure in 50%, inability to advance a stent in 10% and no flow after stent deployment in 10% of the cases. Urgent coronary artery bypass graft surgery was required in 9.7% of the series (10). Therefore, such higher risk TAVR procedures should be performed in larger centers with surgical backup.

## Conclusion

Coronary obstruction is a rare but life-threatening complication following TAVR, especially in patients undergoing aortic VIV that face up to a 6-fold increase of this dreadful event. Understanding the mechanisms involved is a key factor to assess the individual risk of patients through a meticulous pre-procedural evaluation with imaging tools, especially utilizing MSCT, so that additional strategies can be mapped out to avoid or mitigate its consequences. Future studies, to assess novel markers for its occurrence and to potentially evaluate additional treatment modalities, such as the BASILICA technique are warranted, because despite the significant advancements in the TAVR field, with novels techniques and devices, this complication still occurs.

## Author Contributions

FB and HR: conception and design, drafting and revising of manuscript, final approval of the manuscript submitted. JR-C and DD: critical review of the manuscript for important intellectual content, final approval of the manuscript submitted. All authors have participated in the assembly of the current article.

### Conflict of Interest Statement

HR is consultant for Edwards Lifescience and Medtronic Inc. JR-C has received research grants from Edwards Lifesciences, Medtronic, and St. Jude Medical. DD is consultant for Edwards Lifesciences, Medtronic, and St. Jude Medical. The remaining author declares that the research was conducted in the absence of any commercial or financial relationships that could be construed as a potential conflict of interest.
